# A Mechatronic Loading Device to Stimulate Bone Growth via a Human Knee

**DOI:** 10.3390/s16101615

**Published:** 2016-09-29

**Authors:** Sai Krishna Prabhala, Stanley Chien, Hiroki Yokota, Sohel Anwar

**Affiliations:** 1 Department of Mechanical Engineering, Purdue School of Engineering and Technology, Indiana University-Purdue University Indianapolis, Indianapolis, IN 46202, USA; saikprab@iupui.edu; 2 Department of Electrical and Computer Engineering, Purdue School of Engineering and Technology, Indiana University-Purdue University Indianapolis, Indianapolis, IN 46202, USA; schien@iupui.edu; 3 Department of Biomedical Engineering, Purdue School of Engineering and Technology, Indiana University-Purdue University Indianapolis, Indianapolis, IN 46202, USA; hyokota@iupui.edu

**Keywords:** knee-loading, modality, electro-mechanical device, loading force, bone healing, knee rehabilitation, slider crank mechanism, Arduino, Megunolink

## Abstract

This paper presents the design of an innovative device that applies dynamic mechanical load to human knee joints. Dynamic loading is employed by applying cyclic and periodic force on a target area. The repeated force loading was considered to be an effective modality for repair and rehabilitation of long bones that are subject to ailments like fractures, osteoporosis, osteoarthritis, etc. The proposed device design builds on the knowledge gained in previous animal and mechanical studies. It employs a modified slider-crank linkage mechanism actuated by a brushless Direct Current (DC) motor and provides uniform and cyclic force. The functionality of the device was simulated in a software environment and the structural integrity was analyzed using a finite element method for the prototype construction. The device is controlled by a microcontroller that is programmed to provide the desired loading force at a predetermined frequency and for a specific duration. The device was successfully tested in various experiments for its usability and full functionality. The results reveal that the device works according to the requirements of force magnitude and operational frequency. This device is considered ready to be used for a clinical study to examine whether controlled knee-loading could be an effective regimen for treating the stated bone-related ailments.

## 1. Introduction

**Figure 1:**
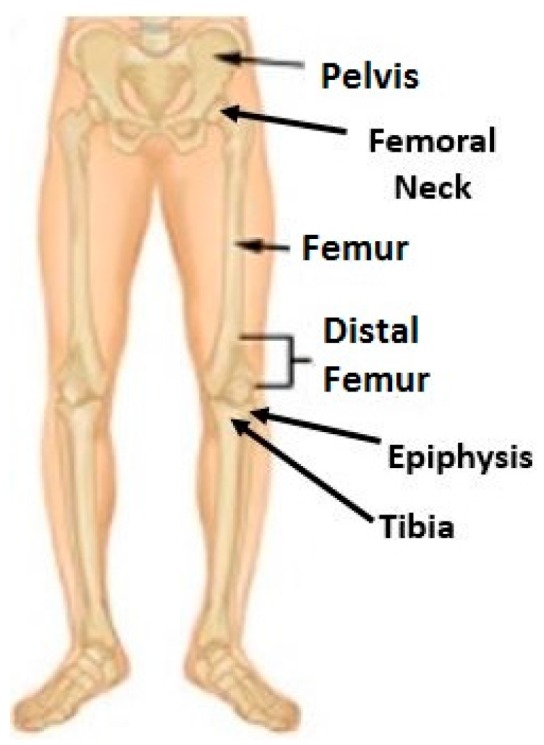
Brief anatomy of lower body bone structure of human body [3].

A bone is a rigid and dense connected tissue which is metabolically active in the body. It is capable of repairing itself and adapting to the subjective stimuli like various physical activities such as walking, swimming, running, etc. The strengthening or weakening of the bone tissues depends on individual’s characteristics like weight, muscle strength, fitness, etc. [1]. When a bone tissue is damaged, it heals over time through the body’s natural healing process. This process can be accelerated by external interference. Mechanical loading effect is one of the procedures to achieve this task under controlled conditions. This innovative modality was validated to possess regenerative capabilities in the areas of distal femur and proximal tibia of the knee bone. It can be achieved by lateral application of force to the knee joint. The effects of the stimulation of bone regeneration are not limited to the applied areas but are seen along the length of the long bone [2]. This is can be explained with the help of the following molecular reaction and biophysical mechanism. When a specific loading force is applied to the epiphyses of the femur and tibia, the trabecular bone tissue, which is characterized by axial stress resistance, resists this force from the opposite direction. This results in deformations in that area. These deformations create a variation of the fluid pressure in the intramedullary cavity. This pressure gradient allows the flow of fluids that carry essential nutrients to the bone cortex initiating osteoblast differentiation and osteogenesis, thus helping in repair and regeneration of the bone tissue [2]. This unique reaction makes this procedure an effective treatment for bone rehabilitation. It helps in reduction of healing time of bone fractures and hastens recovery from bone-related injuries and diseases. The lateral stress application is also less strenuous to the knee bone and reduces the amount of force that needs to be applied to get this result. A human lower body bone structure is shown in Figure 1 [3].

**Figure 2:**
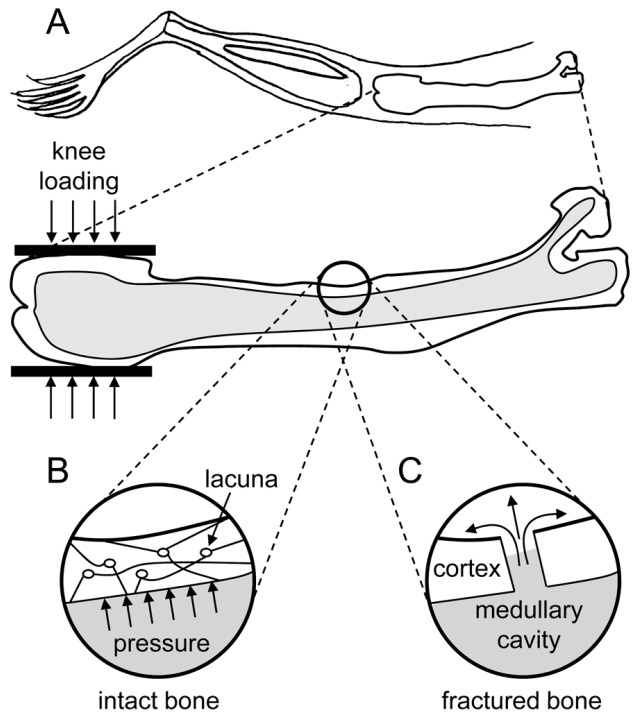
(**A**) schematics of mouse femur under loading; (**B**) pressure increase in the bone cortex; and (**C**) fluid flow in a fractured bone cortex.

The therapeutic effects were primarily observed during the experiments performed on various animals and rodents [4,5]. The effect still needs to be clinically tested on human subjects. The aim of the prototype device presented in this paper is to serve as a test setup for human trials to validate this principle. The most common form of bone injuries are the fractures caused by impact stresses. On average, 6 million people break bones every year in the U.S. [6]. To verify the knee-loading effect, various animal experiments were conducted. One of the important experiments that demonstrated the effects of the knee-loading was the surgical hole recovery experiment on rodents. Approximately 62 female mice were chosen as test subjects and were given surgical holes on their left and right knees under controlled conditions. An experimental mechanical loading setup was used to apply loading force on one of the injured knees, and the other knee was allowed to heal via remote endocrine signaling [7]. During the loading and recovery phases, the samples of the mouse femur were analyzed, and it was found that the frequent knee-loading helped in accelerated healing and closure of the hole on the femur bone rather than the other knee of the mice [4,5,8]. This experiment attests to the effectiveness of the loading phenomenon in reducing healing time, repair and regeneration of the bones. The schematics of the biophysical mechanisms inside the mouse femur and potential fluid flow in the medullary cavity are shown in Figure 2 [1].

In order to evaluate the efficacy of the knee-loading regimen on human subjects, the design of an electro-mechanical device was proposed and developed. The basic idea is to apply controlled direct loads with a specific loading frequency to the knee joint in a lateral direction. Several previous designs for this process were considered as references in choosing the design constraints and requirements for the current design.

## 2. Design Methodology

The design methodology started with figuring out the requirements and constraints of the device. The main requirements for this type of device would be the amount of loading force that it should generate, the frequency of the application of force, the range of motion of the applicator, nature of the actuation source, portability, lightweight quality, and compactness. A prototype device was designed previously with these factors as requirements where a voice coil actuator was used as an actuation source [9]. However, voice coil-based design proved to be bulky and expensive. This paper proposes a new design with considerable modifications, using a less expensive and less heavy brushless DC motor and other control features.

The amount of loading force that the device can produce is a key factor. From previous studies, it was decided that the proposed device should be robust enough to produce different magnitudes of linear force up to a maximum of 40 N. The actual amount of force should depend on an individual’s necessity and will be evaluated by clinical experiments and medical professionals.

The frequency of operation is another key characteristic of the device. This device should be able to operate at different frequencies ranging from 1 Hz to 5 Hz. This means that the device can apply the desired magnitude of loading force at different frequencies. This load–frequency combination will also be evaluated via clinical trials according to patients’ treatment requirements.

The device’s ultimate aim is to serve as a home-based therapeutic apparatus; thus, it needs to be compact in size, lightweight, and portable. For this purpose, the dimensions of the device were constrained to 0.3 m × 0.1 m × 0.2 m. The maximum range of motion of the force applicator of the device was also constrained to 0.012 m. This value was chosen based on the mechanical properties of the human knee like stiffness of bones, muscles and cartilage, so that the applicator does not move too far away from the target area. All design constraints are presented in Table 1.

The actuation source of the device is a brushless DC motor that is capable of rotating at a maximum of 6000 RPM and producing a torque of 1.2 N-m (Anaheim Automation, Anaheim, CA, USA). The maximum speed of the motor is chosen based on the frequency of operation, and the maximum torque was determined from a dynamic simulation of the device.

**Figure 3:**
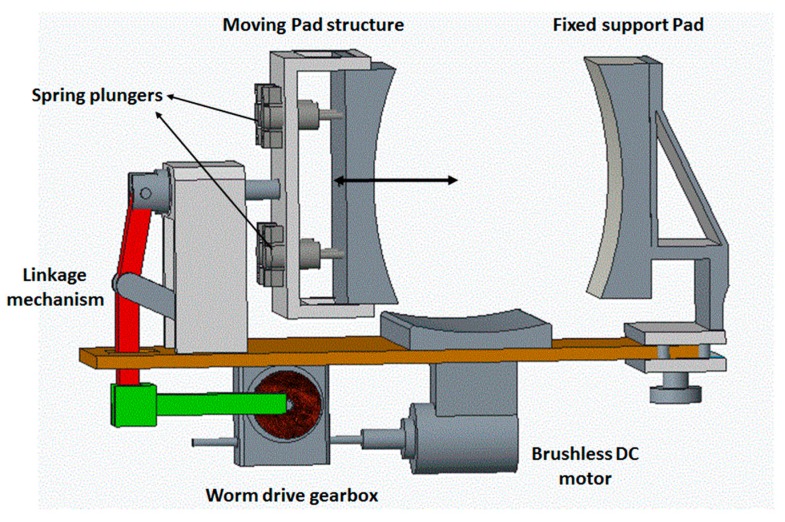
3D CAD model of the knee-loading device.

Considering the average shape and size of a human knee, a 3D CAD model of the knee-loading device was developed using the modeling software PTC Creo Parametric 3.0 (Figure 3) (PTC, Inc., Needham, MA, USA).

The objective of the device is to apply lateral force to the knee by placing the knee between the two pads of the device. The pad on the right is fixed for creating a snug fit to the knee, while the pad on the left applies loading force. The double headed arrow represents the direction of motion of the force-application. The modified slider–crank mechanism with a linear guide block ensures a linear movement of the force-applying pad. In operation, the moving pad pushes the knee against the fixed pad and the springs in the socket are compressed for applying and relieving force on the knee. The distance between the two pads can be adjusted with a knob at the bottom for accommodating knee sizes. The DC motor is mounted at the bottom of the platform. The energy is transmitted to the force applicator through a worm drive gearbox and a modified slider crank mechanism.

### 2.1. Mechanism and Simulations

**Figure 4:**
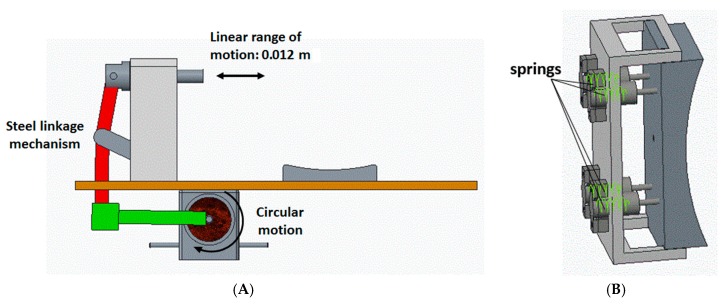
CAD illustrations of (**A**) linkage mechanism of the device; and (**B**) moving pad with spring plungers.

The mechanism employed in the design of the proposed device is a modified slider crank mechanism. It converts the rotary motion given by the worm gearbox to a linear motion. It is connected to a socket that consists of four plungers with springs enclosed in them. These springs ensure that the force-applying pad stays in contact with the knee all the time and also that it provides flexibility and safety of operation. The cumulative spring constant of these springs is 3333.3 N/m. This value is chosen based on the range of motion of the pad. When these springs are compressed fully (as in 0.012 m), a force of 40 N is exerted on the pad. It is illustrated in Figure 4.

The mechanism mainly consists of linkages connected with pin joints. The lengths of the linkage bars are mathematically calculated and then modelled to produce a maximum range of motion of 0.012 m. They are calculated using geometric representations of the mechanism, and, through that, trigonometric equations were deduced to calculate various lengths and angles in the mechanism [10]. Some of them are initially assumed, whereas others are calculated.

The linkage arm was designed using geometrical quantities [10]. Since the range of motion is constrained, the variable quantities can be varied such that the slider gives the required displacement. The slider pin ensures the linear motion of the force-applying pad. It also helps in stabilizing the system as the slider is mounted on a support structure not allowing any compliance. Other mechanical structures were designed, keeping this mechanism as a reference so that the system has overall symmetry like the fixable support pad. The dimensions of the pad are chosen based on an average sized human knee cross-sectional area [10].

The individual forces in the linkages of this mechanism can be evaluated using the dynamic equations given by Newton’s 2nd law. These were obtained with the help of free body diagrams of the mechanism linkages [9]. The governing equations are as follows:

(1) $$F_{12x}-F_{32x}=m_{2}\;{\ddot{p}}_{2x},$$

(2) $$F_{12y}-F_{32y}\;=m_{2}{\ddot{p}}_{2y},$$

(3) $$T_{m}\times N-FR_{l}=\;I_{d}\times \ddot{\theta },$$

(4) $$-F_{32x}+F_{53x}=m_{3}{\ddot{p}}_{3x},$$

(5) $$-\;F_{32y}+F_{53y}\;=m_{3}{\ddot{p}}_{3y},$$

(6) $$F_{32x}d_{3}Sin\theta_{2}\;+F_{32y}d_{3}Cos\theta_{2}-F_{43x}f_{3}Sin\theta_{2}-F_{43y}f_{3}Cos\theta_{2}\;=m_{3}k_{3}^{2}{\ddot{\alpha }}_{3},$$

(7) $$F_{43x}-F_{14x}=m_{4}{\ddot{p}}_{4x},$$

(8) $$-F_{43y}+F_{14y}=m_{4}{\ddot{p}}_{4y},$$

(9) $$-F_{43x}d_{4}Sin\theta_{3}\;+F_{43y}d_{4}Cos\theta_{3}+F_{14x}f_{4}Sin\theta_{3}+F_{14y}f_{4}Cos\theta_{3}\;=m_{4}k_{4}^{2}{\ddot{\alpha }}_{3},$$

(10) $$-F_{53x}+F_{X}=m_{5}{\ddot{p}}_{5x},$$

(11) $$F_{53y}-F_{Y}=m_{5}{\ddot{p}}_{5y},$$

(12) $$F=\frac{1}{12}m_{5}\left(b^{2}+c^{2}\right){\ddot{d}}_{5},$$

where: *F_ijx,y_*: individual force components; *m_n_*: mass of the links; *P_n(x,y)_*: components of the center of mass(G_n_); ${\ddot{p}}_{n\left(x,y\right)}$: acceleration components of the center of mass; *b, c*: width and height of the slider; *d_n_, f_n_*: distances from the center of mass to joints; *k_n_*: radius of gyration; *T_m_*: motor torque; *N*: gear ratio.

**Figure 5:**
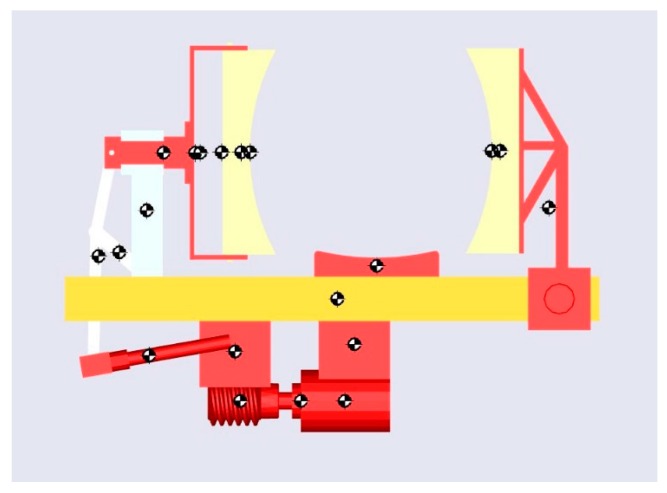
A frame of SimMechanics (version 1.0, MathWorks, Natick, MA, USA) simulated animation of the knee-loading device for proposed design.

These equations are solved numerically using the SimMechanics toolbox (version 1.0, MathWorks, Natick, MA, USA). The SimMechanics is a package in MATLAB (Version R 2014, MathWorks, Natick, MA, USA) that is capable of dynamic simulation of 3D mechanical systems of various applications. It converts 3D CAD models to control blocks and simulates the model in its own environment [11]. This helps in visualizing the dynamics of the system as well. Using this software platform, the device’s CAD model is successfully analyzed and the amount of torque needed to produce the required force from this model was obtained [11]. The SimMechanics environment model is shown in Figure 5 [10].

**Figure 6:**
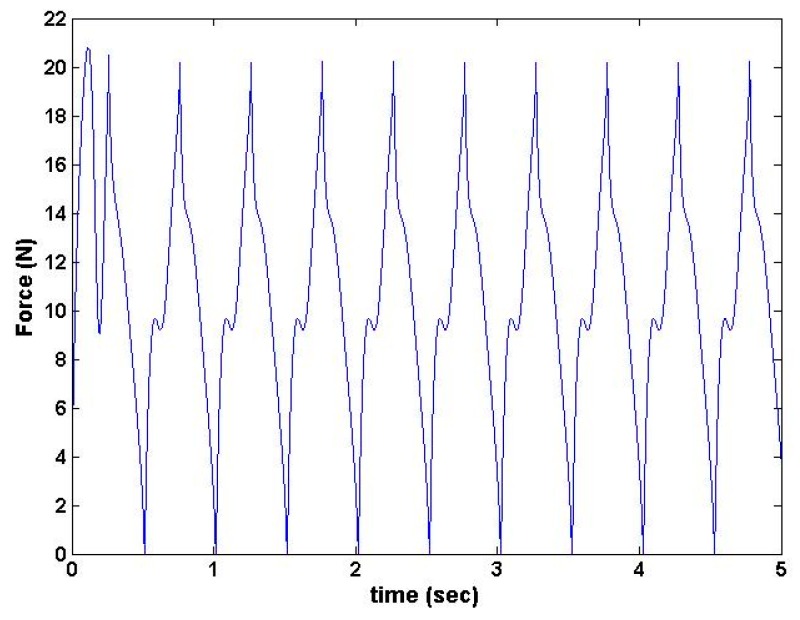
SimMechanics generated waveform showing maximum of 20 N with 2 Hz frequency of the device.

**Figure 7:**
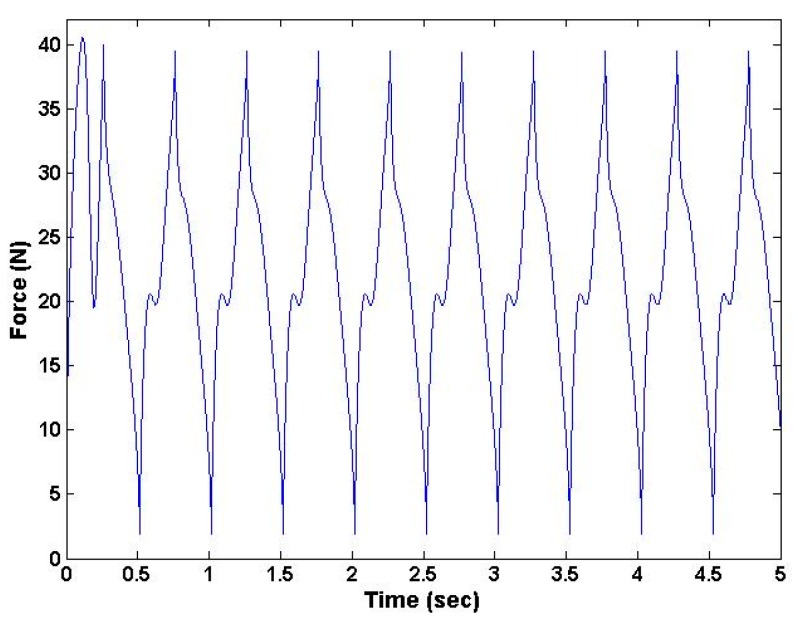
SimMechanics generated waveform showing maximum of 40 N with 2 Hz frequency of the device.

The results from the dynamic simulation from the force sensor blocks in the Simulink toolbox (MATLAB R 2014, MathWorks, Natick, MA, USA ) are shown in Figure 6 and Figure 7 [10]. These results show that the device was capable of producing required loading force and torque at prescribed frequencies.

After a successful dynamic simulation, the CAD model was subjected to static structural analysis to test the structural integrity of the system in ANSYS Workbench 15.0 (ANSYS, Inc., Canonsburg, PA, USA). This analysis is important because the structural strength of the device is a key factor for its durability and effectiveness. Using this analysis, the stresses and deformations on different parts are obtained [12].

**Figure 8:**
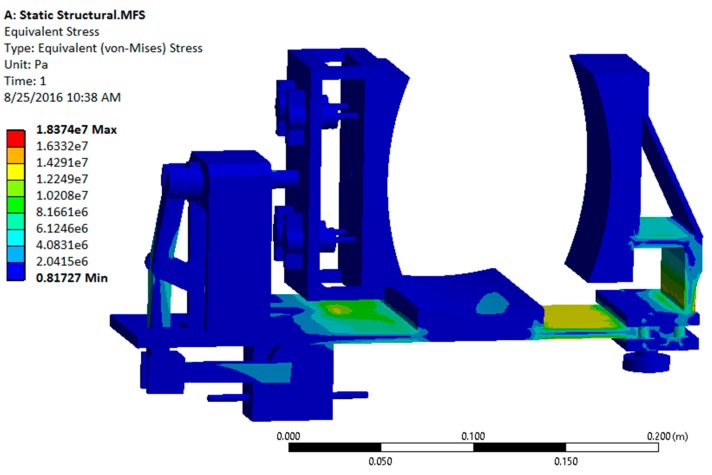
von Mises (equivalent) stress contour plots for all metal configurations.

**Figure 9:**
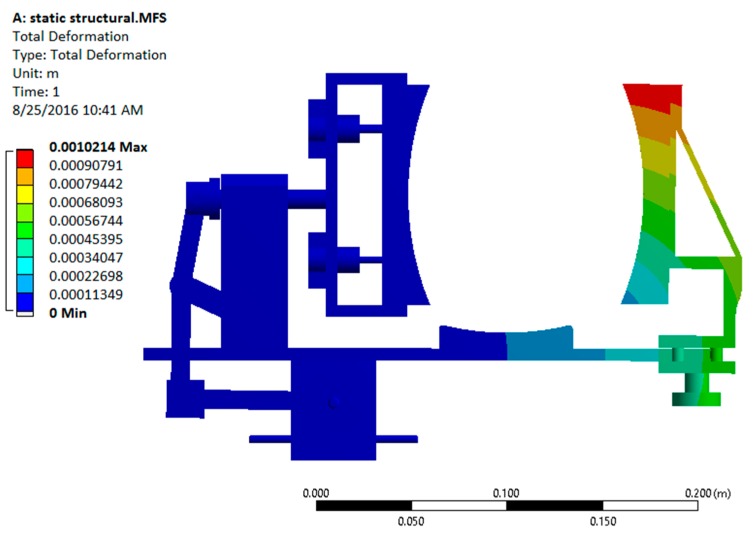
Total deformation plot of the device under the static loading conditions.

Initially, the materials like steel (for the modified slider crank mechanism) and aluminum (for the platform and support structures) were considered to be used in the ANSYS (Analysis System) Workbench. However, static structural analysis showed that the predicted stress and deformation were well within the allowable range. The results from static structural analysis using metallic materials are shown in Figure 8 and Figure 9.

**Figure 10:**
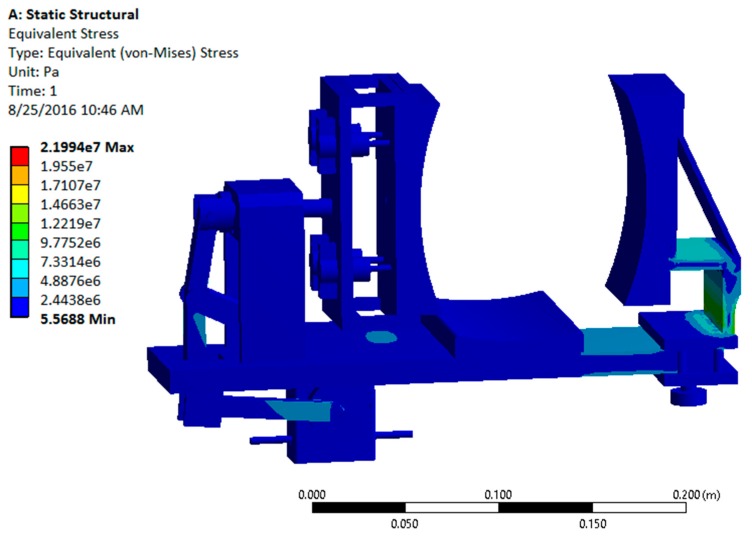
von Mises (equivalent) stress contour plot for metal and Acrylonitrile Butadiene Styrene (ABS) plastic configuration.

**Figure 11:**
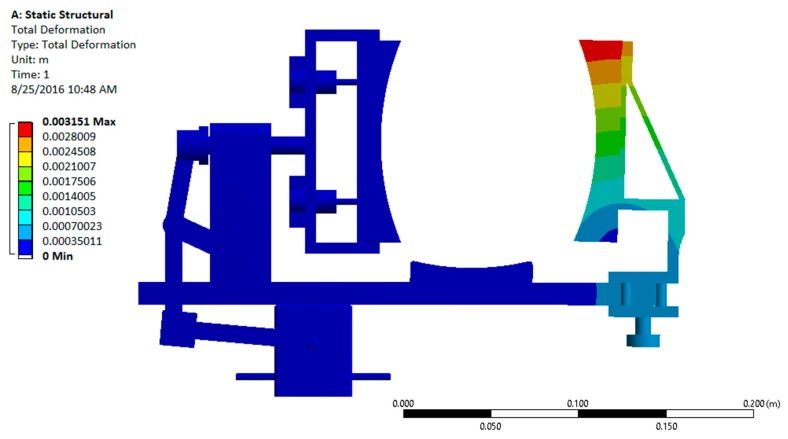
Total deformation plot of the device under the static loading conditions.

The analysis of the design with all metallic components showed that the factor of safety was significantly high (15.1) for the device, indicating that it is over-designed. Since usage of metal would make the device heavy, non-metallic materials such as Acrylonitrile Butadiene Styrene (ABS) plastic were considered. Usage of non-metallic materials would make the device lighter by maintaining a reasonable factor of safety. The revised static structural analysis was performed by replacing aluminum with ABS plastic for the platform and support structures [12]. The results are shown in Figure 10 and Figure 11.

The analysis results show that the stress and deformation on different components are within the allowable range and the factor of safety for this design with the ABS plastic/steel configuration was computed to be 3.6, which is a significant improvement over the all metal design having a factor of safety of 15.1.

### 2.2. Construction of Prototype

The prototype device was built after the structural analysis of the CAD model. The results from the static structural analysis proved that the material choice of steel for linkages, and aluminum for other parts was sufficient for the proof-of-concept study. Some of the components, like spring plungers in the moving pad, were 3D printed, and all other parts were machined using conventional shop tools. The base enclosure was also 3D printed to enclose the DC motor and the gearbox and to provide a stable mounting to the device.

**Figure 12:**
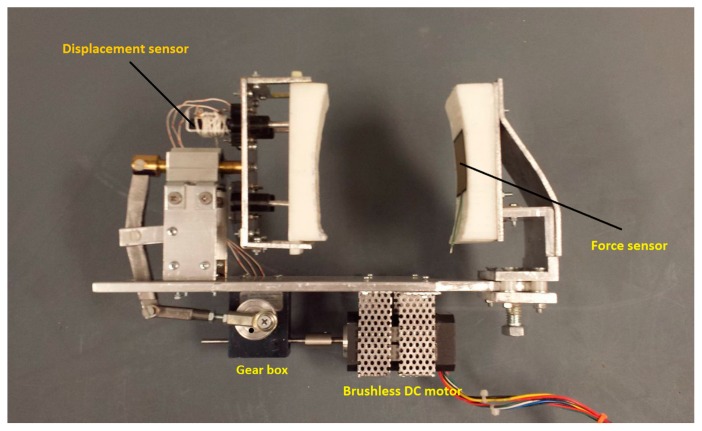
Experimental setup illustrating all the parts of the device.

A suitable brushless DC motor and a speed controller manufactured by Anaheim Automation, Anaheim, CA, USA were chosen as the actuation source and a worm drive gearbox manufactured by SDP/SI (New Hyde Park, NY, USA) was selected. The spring plungers are mounted securely to the back of the socket of the moving pad. The displacement of the force-applying pad directly relates to the amount of force being applied. The displacement is measured by a linear resistance position sensor manufactured by Allied Electronics (Fort Worth, TX, USA). It is mounted at the back of the force-applying pad. A force sensor manufactured by Interlink Electronics (Camarillo, CA, USA) is attached on the fixed pad that senses the amount of force exerted on the pad. The constructed experimental setup is shown in Figure 12.

The loading force of the device can be selected using a variable displacement concept. In order to adjust the applied force, the position of the crank link on the rotating disc (connected to the gearbox) is adjusted. As a result, the range of motion of the force-applying pad is restricted to that corresponding force magnitude. For this prototype, the loading force settings are limited to only four different magnitudes (10, 20, 30, 40 N). To achieve these magnitudes, the position of the crank is changed on the disc to various points that rotate the crank shaft in circles of various radii, resulting in different displacements.

The operation frequency of the device can be controlled by using the speed controller in conjunction with the Arduino microcontroller (Adafruit Industries, New York, NY, USA). The speed controller has an external potentiometer circuit that can change the voltage input to the DC motor. This circuit is controlled by a digital potentiometer circuit that gets its inputs from the Arduino Uno controller. This open source microcontroller is at the heart of all the electronics related to this device. It is an eight-bit open source microcontroller that is very easy and efficient to program [13].

**Figure 13:**
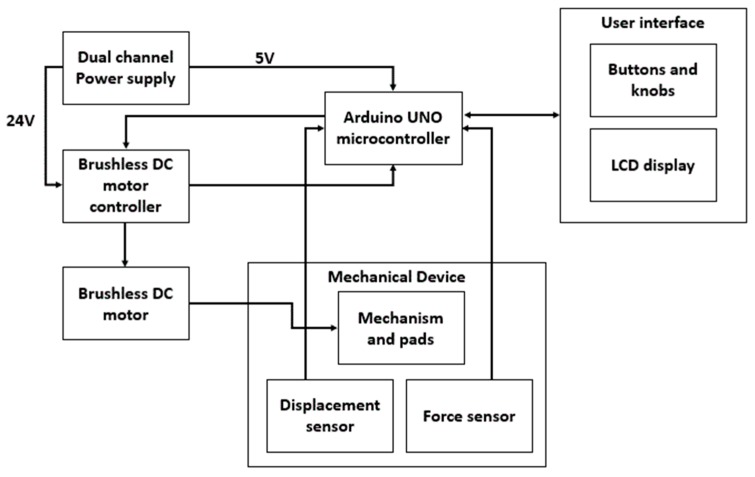
Block diagram of the knee-loading device.

For experimental purposes, a DC-regulated power supply is connected to the setup to power the brushless DC motor and other circuitry. The device also consists of a position sensor to measure the displacement of the force-applying pad and a force sensor to measure the force exerted on the fixed pad. The controller consists of a power switch, emergency device stop button, and frequency and time duration of operation controls in the form of a user-friendly settings layout displayed on an Liquid Crystal Display (LCD). This entire circuitry is enclosed in a 3D printed box that collectively forms the user interface. All the described connections are illustrated in the block diagram of the device shown in Figure 13.

**Figure 14:**
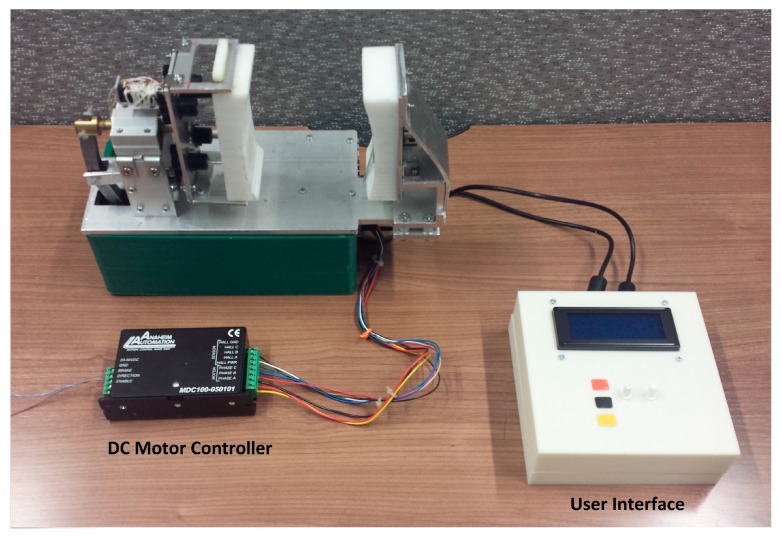
Experimental setup of the knee-loading device.

**Figure 15:**
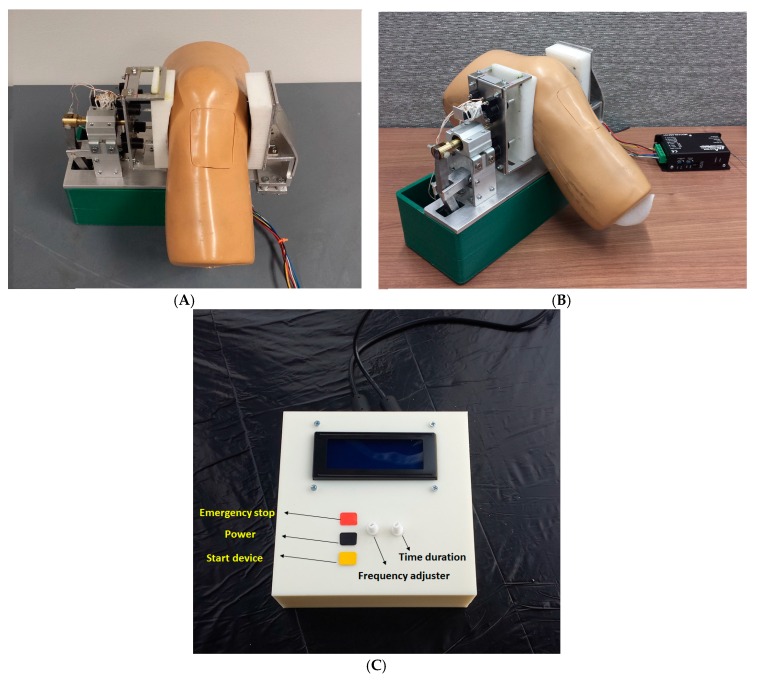
(**A**,**B**) different views of the experimental setup of the knee-loading device; and (**C**) user interface for the knee-loading device.

The final knee-loading device setup for experimental evaluation is shown in Figure 14. A mannequin knee served as the closest replica of an average human knee for the device operation and for evaluating the results of the experiments. The device with the mannequin and the user interface are shown in Figure 15.

The user interface for the knee-loading device is shown in Figure 15C. It consists of an LCD and various circuit boards, with two knobs for selecting loading frequency as well as time duration of a loading session. Furthermore, it contains three buttons for power (black), start (yellow) and emergency stopping (red) the device.

### 2.3. Operational Procedure

In order to use the device, the adjustable pad on the right can be adjusted and the knee is placed in between the two pads, and this pad is to be tightened so that the knee has a snug fit. The controller and the device are powered ON subsequently. The force needed is chosen before using the device and the position of the disc is adjusted accordingly. The frequency and the time duration of force application were selected using the user interface, and the device was started. The device can be stopped at any time using the emergency stop button. After the selected time duration, the device stops.

## 3. Results and Discussion

In order to display the achieved magnitude of displacement of the moving pad and the magnitude of force exerted on the knee, the Arduino controller is interfaced with Megunolink software, which is a Windows application for sending and receiving serial data (Version 1.5.16073.0313, F6 Digital Media, Hamilton, New Zealand). It is capable of converting serial data coming from the sensors to waveforms that can be easily interpreted. It uses a modified C language with dedicated commands, functions and libraries, similar to Arduino programming language [14].

**Figure 16:**
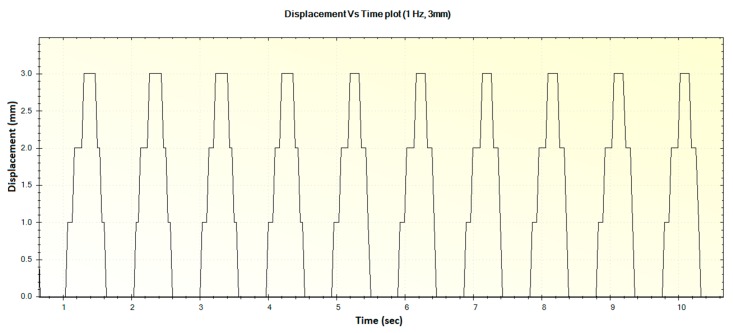
Generated waveform for 1 Hz operation producing a displacement of 3 mm.

**Figure 17:**
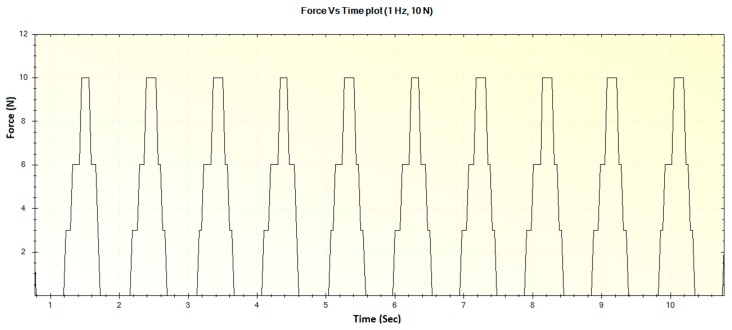
Generated waveform for 1 Hz operation producing a force of 10 N.

**Figure 18:**
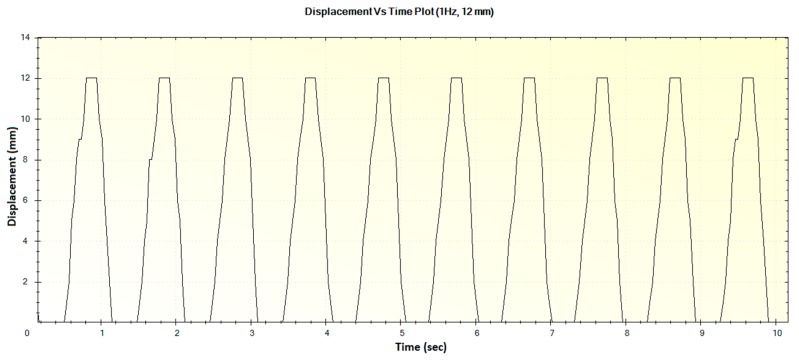
Generated waveform for 1 Hz operation producing a displacement of 12 mm.

**Figure 19:**
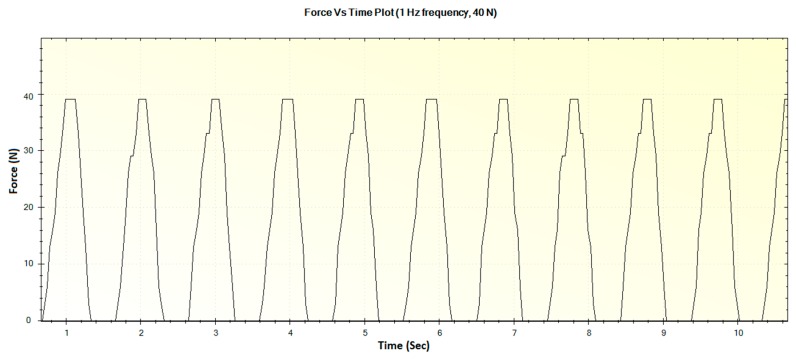
Generated waveform for 1 Hz operation producing a force of 40 N.

**Figure 20:**
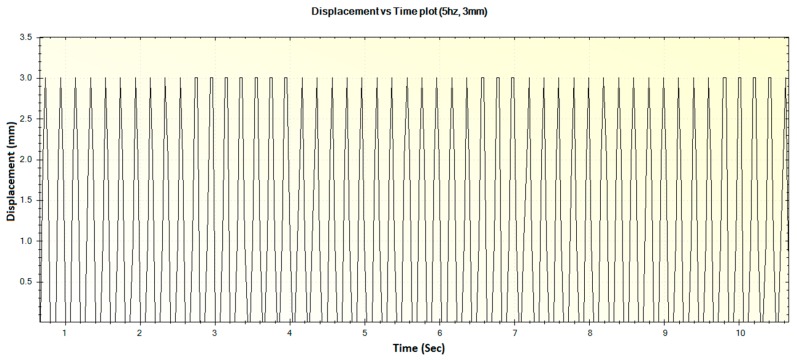
Generated waveform for 5 Hz operation producing a displacement of 3 mm.

**Figure 21:**
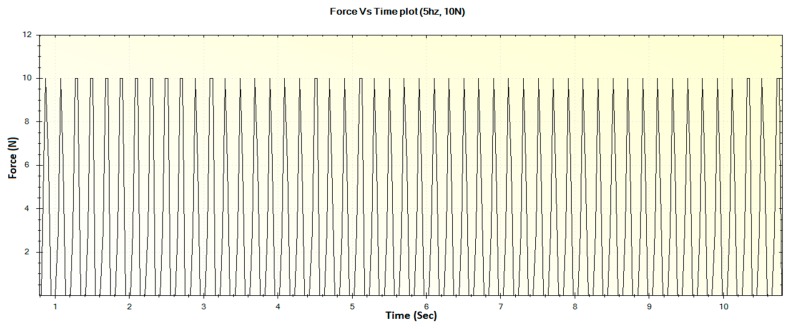
Generated waveform for 5 Hz operation producing a force of 10 N.

**Figure 22:**
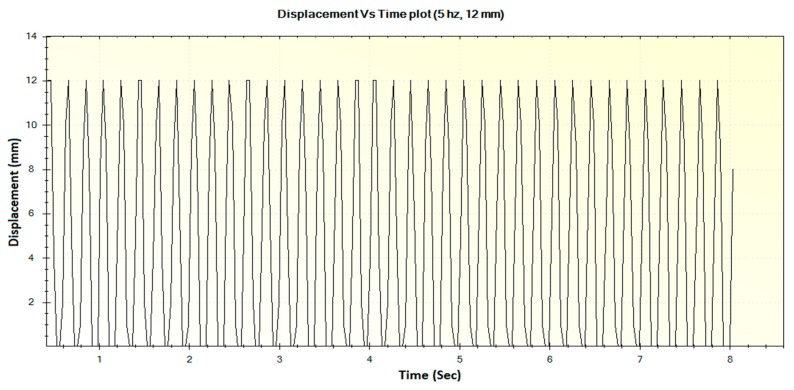
Generated waveform for 5 Hz operation producing a displacement of 12 mm.

**Figure 23:**
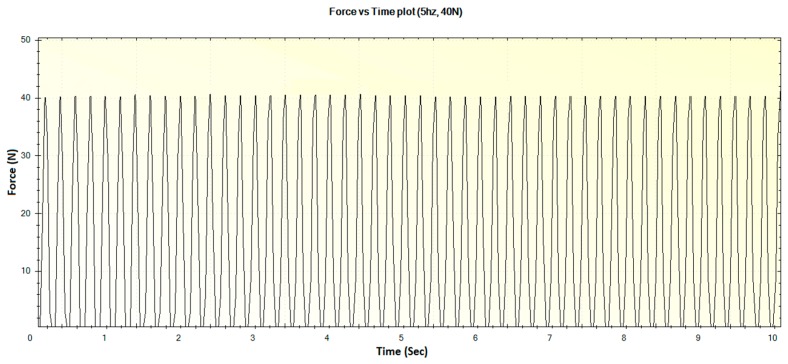
Generated waveform for 5 Hz operation producing a force of 40 N.

In this section, the experimental results such as various displacements, frequencies of operation and magnitude of forces are illustrated in Figure 16, Figure 17, Figure 18, Figure 19, Figure 20, Figure 21, Figure 22 and Figure 23. These were taken with the help of the Megunolink software interface. The results with these waveforms verify that the real-time operation of the device is feasible as required.

In Figure 16, the step-like behavior of the device at certain points can be attributed to possible friction in the moving pad spring socket. As the spring plungers were 3D printed with ABS plastic, some amount of frictional force resists the smooth motion of the pad during low frequency operation. However, it was observed that the device was not stuck or stalled during such operation, and this pattern was also not observed during higher frequencies.

The corresponding force versus time plot for the 1 Hz frequency with a maximum displacement of 3 mm is shown in Figure 17.

For an operation frequency of 1 Hz, the generated displacement of 12 mm is shown in Figure 18. The corresponding force versus time plot for the 1 Hz frequency with a maximum force of 40 N is shown in Figure 19.

In a similar fashion, the other displacements and corresponding force waveforms can be defined. For an operational frequency of 5 Hz, the generated displacement of waveform with maximum displacement of 3 mm is shown in Figure 20. The corresponding force versus time plot for the 5 Hz frequency is shown in Figure 21.

For an operational frequency of 5 Hz, the generated displacement of waveform with maximum displacement of 12 mm is shown in Figure 22. The corresponding force versus time plot for the 5 Hz frequency is shown in Figure 23.

It is clear from the experimental observations above that the built prototype device was able to meet all of the design requirements as set forth from a usability and functionality standpoint. However, the current design on the device is only capable of applying four different magnitudes of loading force. In addition, the weight of the device could be further reduced by using 3D printed ABS plastic parts instead of certain metal parts like the platform and support structures. The presence of stiction (Static Friction) in the 3D printed spring plungers can be reduced by using lubricated and sealed spring plungers.

## 4. Conclusions

This paper presents an innovative device that applies the desired knee-loading forces at desired frequencies of operation that are aimed at treating the stated bone-related ailments. Its design was improved from the previously built prototypes, with an added capability of providing linear force at desired frequencies. With success treating such bone ailments in prior animal studies, the knee-loading efficacy on humans using this device can be evaluated experimentally through clinical trials.

The results in this study show that the device works according to the requirements of loading force and operational frequency. By varying a crank position on the disc and selecting different frequencies with the user interface–microcontroller, the desired frequency and loading force can be achieved.

The prototype device is constrained to the predetermined dimensions, and it satisfies the compactness requirement. The device is mostly made of metallic materials. When the device was analyzed in ANSYS Workbench, however, the result showed that some of the structural components could be replaced with ABS plastic material.

## Figures and Tables

**Table 1:** List of design constraints for the proposed device.

Design Constraint	Value	Units
Maximum Amount of loading force	40	N
Frequency of application	1 to 5	Hz
Range of motion of the applicator	0.012	m
Dimensions of the device	Length: 0.3	m
	Width: 0.1	
	Height: 0.2	
Weight of the device	<3.5	kg
DC (Direct current) motor max. Speed	6000	RPM
Actuator required torque	1.2	N-m
